# Locally-Procured Fish Is Essential in School Feeding Programmes in Sub-Saharan Africa

**DOI:** 10.3390/foods10092080

**Published:** 2021-09-02

**Authors:** Molly B. Ahern, Shakuntala Haraksingh Thilsted, Marian Kjellevold, Ragnhild Overå, Jogeir Toppe, Michele Doura, Edna Kalaluka, Bendula Wismen, Melisa Vargas, Nicole Franz

**Affiliations:** 1 Fisheries and Aquaculture Division, Food and Agriculture Organization of the United Nations (FAO), 00153 Rome, Italy; Nicole.franz@fao.org; 2 WorldFish, Penang 11960, Malaysia; s.thilsted@cgiar.org (S.H.T.); b.wismen@cgiar.org (B.W.); 3 Institute of Marine Research, 5817 Bergen, Norway; marian.kjellevold@hi.no; 4 Department of Geography, University of Bergen, 5007 Bergen, Norway; Ragnhild.Overa@uib.no; 5 Sub-Regional Office for Mesoamerica, Food and Agriculture Organization of the United Nations (FAO), Panama City 32408, Panama; Jogeir.toppe@fao.org; 6 School-Based Programmes Unit, World Food Programme, 00148 Rome, Italy; michele.doura@wfp.org (M.D.); edna.kalaluka@wfp.org (E.K.); 7 Nutrition Division, Food and Agriculture Organization of the United Nations (FAO), 00153 Rome, Italy; melisa.vargas@fao.org

**Keywords:** home-grown school feeding, food and nutrition security, fish, fish products, sub-Saharan Africa, small-scale fisheries, fish processing, child nutrition, adolescents, SDG1, SDG2, SDG3, SDG14

## Abstract

Fish make an important contribution to micronutrient intake, long-chained polyunsaturated omega-3 fatty acids (n-3 LC-PUFAS), and animal protein, as well as ensuring food and nutrition security and livelihoods for fishing communities. Micronutrient deficiencies are persistent in sub-Saharan Africa (SSA), contributing to public health issues not only in the first 1000 days but throughout adolescence and into adulthood. School feeding programs (SFPs) and home-grown school feeding programs (HGSF), which source foods from local producers, particularly fisherfolk, offer an entry point for encouraging healthy diets and delivering essential macro- and micronutrients to schoolchildren, which are important for the continued cognitive development of children and adolescents and can contribute to the realization of sustainable development goals (SDGs) 1, 2, 3, 5, and 14. The importance of HGSF for poverty alleviation (SDG1) and zero hunger (SDG 2) have been recognized by the United Nations Hunger Task Force and the African Union Development Agency–New Partnership for African Development (AUDA-NEPAD), which formulated a strategy for HGSF to improve nutrition for the growing youth population across Africa. A scoping review was conducted to understand the lessons learned from SFPs, which included fish and fish products from small-scale producers, identifying the challenges and best practices for the inclusion of fish, opportunities for improvements across the supply chain, and gaps in nutritional requirements for schoolchildren which could be improved through the inclusion of fish. Challenges to the inclusion fish in SFPs include food safety, supply and access to raw materials, organizational capacity, and cost, while good practices include the engagement of various stakeholders in creating and testing fish products, and repurposing fisheries by-products or using underutilized species to ensure cost-effective solutions. This study builds evidence of the inclusion of nutritious fish and fish products in SFPs, highlighting the need to replicate and scale good practices to ensure sustainable, community-centred, and demand-driven solutions for alleviating poverty, malnutrition, and contributing to greater health and wellbeing in adolescence.

## 1. Introduction

Animal-source foods (ASFs), including fish and other aquatic foods, dairy, eggs, meat, and poultry are nutrient-rich and particularly important for the diets of children and vulnerable populations [1]. Fish is rich in multiple micronutrients (vitamins A and B12, iron, and zinc) and is one of few natural dietary sources of vitamin D3, iodine, and essential fatty acids, including eicosapentaenoic acid (EPA) and docosahexaenoic acid (DHA), which are often lacking in the diets of vulnerable populations. It also enhances the absorption of micronutrients such as iron and zinc from plant-source foods when consumed together [2].

Deficiencies in vitamin A, zinc, iron, and iodine are persistent public health problems across SSA, contributing to iron-deficiency and anaemia, night blindness, thyroid disorders, and diarrheal deaths [3,4,5,6]. Many efforts to improve nutrition focus on the first 1000 days of life, with evidence of positive growth outcomes have been associated with the incorporation of fish into the diets of mothers, infants, and young children [7,8,9,10,11]. However, most recently, the State of School Feeding Worldwide has highlighted the urgent need for a paradigm shift to expand the “age-siloed” approach to child health and development beyond the first 1000 days to embrace children’s needs across the life cycle (the first 8000 days) [12]. These additional 7000 days are evidenced as a period of continued growth and development, and link two periods which are often of focus for public health nutrition—from conception to two years of age and during the reproductive age (15–49 years old) [13,14,15,16]. Children and adolescents usually spend much of this time in schools, offering an entry point to improve nutrition.

Proper nutrition is particularly important for adolescent girls in countries where early marriage and teenage pregnancies are common, to address the intergenerational cycle of malnutrition, evidenced through low birthweight and poor foetal growth patterns [17]. Brain development is not completed by the age of two years, as the frontal lobes of the brain develop in “spurts” from birth to two years, between ages seven and nine, and again in the mid-teenage years [18]. Evidence indicates that malnourished children have higher absentee rates and poorer performance due to illness, have poorer brain development and cognition, or are less ready for school [19]. Iron deficiency affects 300 million schoolchildren globally, causing a loss of six IQ points per child [12]. There is evidence of iodine deficiency being a main cause of brain damage in childhood [20], as well as of iron and omega-3 fatty acids positively influencing brain development [21,22,23]. Deficiencies in DHA expose children between the ages of seven and nine to impaired brain development during this “brain spurt” [18], potentially compromising intellectual development, academic performance, memory and learning [23,24]. Randomized control trials with children of pre-school and kindergarten age [25,26], as well as school-aged children of eight to nine years old [27] and 14–15 year-olds [28] demonstrated a positive impact on nutritional status, while the results on cognition associated with the consumption of fatty fish are not conclusive.

At the beginning of 2020, one in every two schoolchildren (388 million) received school meals in 163 countries [12]. However, by April 2020, widespread school closures due to the COVID-19 pandemic deprived 370 million of those children of daily meals [12]. In pre-COVID-19 times, two-thirds of countries in Africa, the Americas, and Southeast Asia provided school meals [19]. School feeding programs (SFPs) have been used for decades to provide many benefits to children, their families, and their communities. They can work as a social safety net for low-income households, address equity issues and “level the playing field”, increase school enrolment rates and reduce absenteeism (especially for adolescent girls) in the short term [24,29,30]. Improving nutritional status, cognitive development, and educational outcomes through the retention of students and sustained attendance are longer-term goals [24]. However, SFPs in many developing countries lack guidance on nutrition and food safety standards, as well as menu composition, which may result in a misalignment with the nutritional requirements of targeted schoolchildren and adolescents [31]. SFPs are constrained by little or unstable funding, technologies, and infrastructure while having many students to feed, resulting in low-quality meals and filling but nutrient-poor foods, and ASFs may be lacking or completely excluded [19]. The need for nutrient-dense ASFs to be included in school menus is apparent, as they have been evidenced to improve growth, cognition, and behavioural outcomes, in comparison to meals that are predominantly plant-based [32,33,34,35,36,37,38]. These outcomes have been linked to a greater intake of vitamin B12, iron, and zinc through consumption of ASFs while also increasing iron and zinc absorption from fibre and phytate-rich plant-source staple crops [32,39,40,41]. The majority of these studies, however, looked at the role of meat, eggs, or dairy in SFPs, but excluded fish.

The Human Capital Index for Africa puts the region at 40 percent of its potential, with a projected growth in GDP if goals for health and education are achieved [12]. The most important phase for human capital development is the first 8000 days of life [42]. Africa’s growing youth population and high prevalence of early childbearing [43] highlight the urgent need for strategies such as SFPs for improved nutrition throughout adolescence. Although average fish consumption across Africa is lower than the global average (almost 10 kg/capita/year in comparison to 20.5 kg/capita/year), it accounts for a much greater percentage of animal protein in diets in Africa, as it is often the most consumed animal-source food. Official statistics from the FAO show that fish accounts for more than 50% of ASF in the diets of coastal populations in countries such as Sierra Leone, Gambia, Mozambique, Cameroon, and Ghana (with other studies evidencing as high as 80%) [44,45]. In countries with inland water bodies such as Zambia, Malawi, and Uganda, fish accounts for 30–40% of dietary animal protein (these figures are based on apparent consumption, which is not equal to actual dietary intake) [45]. These figures demonstrate that fish, and particularly small fish, which are often dried and consumed whole [9], are the most accessible ASF, particularly for poor communities in SSA. Projections for 2030 show that per capita fish consumption will increase in all regions except Africa due to a rising population, contrasted by the slow development of aquaculture on the continent [46]. However, there is much evidence that catches from African inland fisheries are underreported and underestimated, and there is potential to increase consumption of freshwater fish if direct human consumption is prioritized [47].

SFPs are often aimed at educational goals, such as increasing enrolment and decreasing absenteeism; however, SFPs can also achieve non-educational goals, including providing livelihood opportunities for local producers, traders, caterers, and other local stakeholders, including support to rural women’s livelihoods and incomes [48]. When SFPs source foods locally from smallholder farmers, it is called home-grown school feeding (HGSF) [48]. HGSF can be seen as a channel to stimulate local economies and livelihoods by providing a market and regular source of income for small-scale food producers, while also ensuring that a variety of fresh and nutritious foods are included in school menus [31], thus contributing to an accelerating progress towards achieving sustainable development goals (SDGs) such as zero hunger (SDG 2) and poverty eradication (SDG 1). Depending on the objectives and implementation models, HGSF can also contribute to other SDGs in the 2030 Agenda for Sustainable Development, including SDGs 3, 4, 5, 8, 10, 14, 15, and 17 [49]. The integration of fresh or perishable foods, such as fish, in SFPs and HGSF are often disregarded based on logistic or hygienic concerns such as processing, preservation, and storage. These concerns prevail despite the current practice in SSA of fish supply chains reflecting that the majority of fish (particularly the more affordable small fish) are distributed and consumed in dried or smoked form [9,50].

Policies in low- and lower-middle-income countries (LMICs) have been driven by the need to reduce poverty, establish social safety nets for vulnerable households, and increase the educational attainment of the population, especially for primary school children [31]. Increasingly, low- and lower-middle-income countries commit to implementing HGSF programs with the aim of stimulating local food production, markets, and economies while incorporating locally produced foods into the diets of school children [31]. Rigorous trials have demonstrated both the economic and non-economic benefits from SFPs and HGSF, although the cost-per-meal and funding varies [12]. In most middle-income countries, national budgets support school feeding programs, whereas investments and support are still needed in low-income countries, where the cost of the meal, although relatively low, may represent a larger proportion of education costs [12]. Recognizing the need to enhance child nutrition and support local producers, the New Partnership for Africa’s Development (NEPAD), now African Union Development Agency–NEPAD (AUDA-NEPAD), recently made HGSF one of their flagship programs under the food and nutrition security program [51]. Despite the good intentions of these commitments, these programs may be chronically underfinanced and there is little known evidence of the inclusion of locally procured fish in SFPs.

Although SFPs are often led by the education sector [52], the integration of health, nutrition, livelihoods, agriculture, environment, and food production requires the engagement and coordination of various stakeholders in the food system, including public and private actors and civil society, to reshape food production and post-harvest supply chains to meet nutritional needs, while also considering the socioeconomic and environmental drivers and feedbacks in the system, including social and environmental welfare [10,53,54]. A sustainable food system has to be equitable and inclusive, ensuring the production, processing, and distribution of good quality, safe, acceptable, and affordable food, reducing food loss and waste, and that food system actors earn enough money to live free from poverty, both now and in the future [53,54]. The food system provides many entry points for HGSF and, at the same time, can strengthen food systems in supporting small-scale producers and organizations through a stable demand for locally procured products, improved food safety and quality, and the provision of social protection to vulnerable populations [48].

## 2. Materials and Methods

A scoping review [55] using the methodologial framework developed by Arksey and O’Malley [56] was used to identify evidence from identified studies, reports, and pilot programs including fish in SFPs. Research questions around the source and form of fish utilized in SFPs were included to explore the successes and challenges in the fish supply chain, including production and processing and how these shape access, affordability, food safety and preferences.

- Where have fish or fish products been included in SFPs or HGSF in SSA (particularly including locally procured fish)?
- What are the forms of fish or fish products used in HGSF (fresh, frozen, dried, smoked, value-added products): whole fish (including bones, eyes, viscera) or only muscle tissue/fillet?
- What were the challenges or lessons learned from these programs, and what are the opportunities for improved nutrition and health outcomes of HGSF including fish?

**Figure 1:**
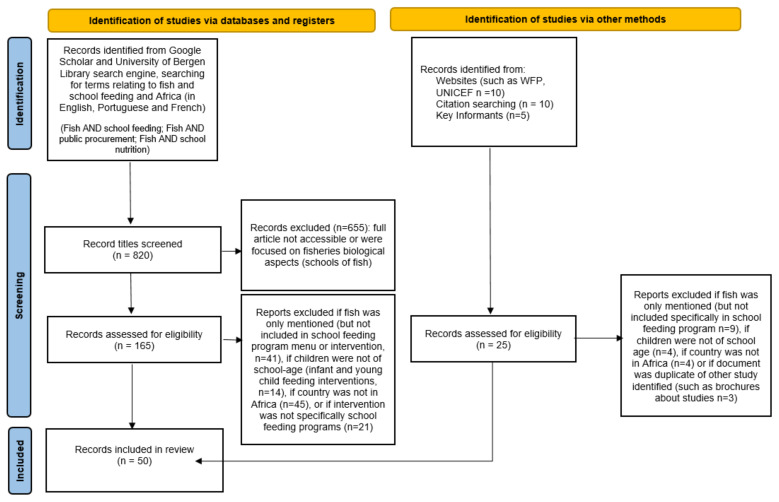
Search strategy and search terms.

Two electronic databases (Google Scholar and the University of Bergen Library search engine) were used to identify literature. The literature search was conducted by the lead author between May and August 2020, and the search was replicated in May–June 2021 to validate initial results and assess if any additional literature qualified for inclusion prior to submisssion for publication. Figure 1 displays the search strategy, eligibility criteria, number of articles included and excluded, and the process of consultation with informants from key institutions involved in school feeding and fisheries, such as the World Food Programme (WFP), the Food and Agriculture Organization of the United Nations (FAO), and the WorldFish Center. Search terms were initially used in the English language; however, key informants noted that examples of SFPs in Africa may be described in other languages including French or Portuguese. No limitation on the publication date of studies was included, as a key informant informed the lead author that fish protein concentrate (FPC) was used in SFPs since the 1950s; however, few documents from before the 1970s were publicly accessible through search engines. The results in this review include evidence from SFPs in SSA, and are not intended to be an in-depth systematic review but rather to provide a broad summary of available evidence from journal articles and grey literature, such as impact evaluations and program reports and summaries of SFPs including fish and fish products.

**Figure 2:**
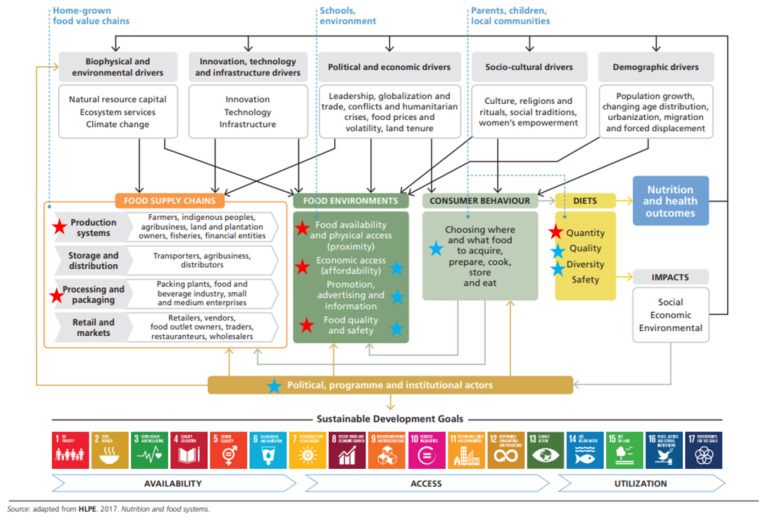
Food systems framework with entry points for HGSF [47] (Reproduced with permission from FAO and WFP, Rome, Italy), with identified challenges marked with red stars and good practices with blue stars. Note: Economic access and food quality and safety were highlighted as challenges in some records, while good practices were also identified in relation to these.

The results present the various challenges and opportunities of implementing SFPs and particularly HGSF programs within the food systems framework (Figure 2). The discussion attempts to further analyze challenges and opportunities to replicate and scale the best practices for the inclusion of fish in SFPs, looking at food production; food handling, storage and processing; food trade and marketing; consumer demand, food preparation and preferences, and broader impacts (social, environmental, and economic), as well as the various drivers that influence the food system.

## 3. Results

### 3.1. Description of SFPs including Fish

A total of 49 articles, program reports and summaries, impact evaluations, and pilot studies including fish in SFPs were identified from 16 (out of 46) SSA countries. The dates of publications included in this review ranged from 2001 to 2021 (see Appendix A), although it is noteworthy that records of fish included in SFPs in non-SSA countries (such as Chile, Mexico, and Thailand) dated as far back as 1954 [57], demonstrating that the inclusion of fish in SFPs has been considered for many decades. Table 1 summarizes the evidence of SFPs including fish, and Appendix B gives more detail about the source and form of fish included in each study. SFPs included in this review were financed through various mechanisms—some were fully implemented by the national government, some were implemented through collaboration between WFP, non-governmental organizations (NGOs) and the national government, and others were financed or co-financed by parents and community donations of money, food, or other necessary items (fuel wood, etc.). Seventeen (n = 17) records stated that fish was sourced from small-scale producers (fisheries or aquaculture). The degree of focus on fish within each record varied, from only one mention of fish in the SFP menu to the specific focus on the development, testing, and supply-side interventions for ensuring a sustainable supply of acceptable and safe fish products for SFPs.

### 3.2. Challenges and Good Practices for Inclusion of Fish in SFPs and HGSF

Challenges in the inclusion of fish in SFPs and HGSF programs were identified in food supply chains, food environments, and the meeting of quantitative recommendations for diets, while good practices were identified in food environments, consumer behavior, and evidence of fish in SFPs contributing to improved dietary quality and diversity.

#### 3.2.1. Challenges in Food Supply Chains

Food supply chains refer to the activities and actors that take food from production to consumption and disposal, and involves many stakeholders, including large- and small-scale actors (both public and private sector), driven by external factors such as biophysiology, environment, innovation, technology, and infrastructure. The decisions made by one group of actors at one stage of the supply chain affects others, and these decisions influence the way food is produced and processed along the supply chain [108].

##### Production Systems: Local Fishers, Fish Processors, and Their Organizations

A constant and sustainable supply is necessary to ensure that fish and fish products are included into HGSF. Local small-scale producers, processors, and distributors are key stakeholders in making fish and fish products available and accessible to school kitchens. One study has identified the local procurement from small-scale producers and their organizations as a challenge, as local small-scale fishers and fish processors may be informally organized and may not be familiar with food quality standards [63]. The same study recommended several strategies, including raising awareness about objectives, reinforcing accountability, and allowing for different types of contributions (money, goods, or services) from stakeholders (government, parents, sponsors) to support HGSF programs and ensure continuous supply from local producers [63]. Furthermore, measures to improve small producers’ access to markets, and capacity building for organizational development to strengthen their ability to plan for production and processing, are necessary to ensure that the supply of food to HGSF programs are not disrupted or terminated [62].

##### Processing

Fish harvested or captured from wild sources are often processed and packed, either through formal processing plants or informal small-scale processors, including women cooperatives and home-based processors. The infrastructure, technologies, and methods for processing vary across communities, but the most common product in the SSA region are dried or smoked fish. The dehydration of fish enhances the nutrient density, enables longer storage life and eases the transportation of fish and fish products across greater distances. Few studies noted that schools lacked infrastructure and equipment for fish preservation, and one study noted that workload of school cooks increased in school canteens due to increased processing or preparation of fish [63]. Few studies included fish protein concentrate (a bland-tasting powder produced from fish, involving a step of extraction, with higher concentrations of protein than the raw material) [109], fish powder (powder produced from grinding whole fish) [70,71,72,101], or tuna frame powder (powder produced from grinding bones or frames of larger fish) [66,67]. Most of these studies utilized hammer mills to grind whole small fish or fish by-products such as tuna frames into powder; however, in all three examples, the fish processing was completed in pilot plants connected to research laboratories [66,67,106].

#### 3.2.2. Challenges in Food Environments

The food environment refers to the physical, economic, political, and sociocultural context in which consumers engage with the food system to make their decisions on acquiring, preparing, and consuming food. The key elements identified that may influence consumer behaviour towards food acquisition are: Food availability and physical access; Economic access; Promotion, advertising, and information; Food quality and safety.

##### Food Availability and Inadequate Quantity in Diets

Access to fish and fish products may be greater for populations located closer to water bodies, and schools located further away from water bodies may be less inclined to include fish in HGSF. Supply and access to fish were cited as challenges in a couple of studies [63,88,91,92]. Three studies in Nigeria [88,91,92] noted rather small quantities of fish in school meals, one included a cost–benefit analysis showing that school meals including 2–4 g of fish on a dry weight basis were nutritionally adequate for the cost [88], while another discussed the low zinc content of meals possibly linked to inadequate quantities of fish included [92]. However, fish was not always included in meals due to issues with regular supply [88].

##### Affordability and Cost

The economic cost of fish may be another limiting factor for their consistent inclusion in HGSF programs. Four records indicated that the cost of school meals including fish (and other ASFs) was as a challenge [63,73,88,105], two of which noted that fish is either supplied in canned form or through aquaculture [73,88], while a key informant informed the author that fish fillets were used in the third study (in Cabo Verde) [63]. In Cabo Verde, the inclusion of fish in school meals raised the cost by 20%, although it is not noted how this has impacted consistent feeding of children or inclusion of fish. In Togo, local groups of women responsible for cooking (“femmes-mamans”) were allotted an amount of USD 0.31 per child, which was noted as insufficient to afford adequate amounts of meat or fish, as cost varied due to several factors including distance to food markets, seasonal price variations, and regional fluctuations in food prices [105]. A lack of monitoring of school meals meeting minimum standards was linked to reported differences in meal composition, as some groups of women were found to reduce amount per serving of the more expensive ingredients (often meat or fish) and served larger portions of staple foods to increase their profits [105].

##### Food Quality and Safety

Poor infrastructure, including road access, cold chains, and infrastructure for monitoring nutrition and food safety standards, limits the distribution of perishable food items, and may compromise the quality and safety of fish for inclusion into school meals. Two studies, one in Angola and another in Sao Tome and Principe, identified the lack of cold chains as a challenge for integrating fish into SFPs [58,96]. In the study in Sao Tome and Principe, the local fishermen and saleswomen association requested financial help to purchase a freezer for the school, an initiative which was accepted and funded by various stakeholders involved [96]. In Togo, the study noted that quality and quantity assurance for school meals are necessary in order to ensure that school lunches meet the minimum meal standard requirements [105].

#### 3.2.3. Good Practices: Multistakeholder Partnerships between Political, Programme and Institutional Actors

Multistakeholder partnerships (including ministries of education, health, nutrition and agriculture, local small-scale fisherfolk, parents, school personnel, and children themselves) were proven to be essential for continuity of the intersectoral work of SFPs, as well as for the ownership of different activities by different government agencies and community members [62,96,107]. This approach was proven to maximize impact and reduce costs by generating synergies and opportunities for dialogue amongst the various stakeholders.

#### 3.2.4. Good Practices in Supply Chains

In Cabo Verde, it was noted that supply models needed to be adapted for each school and the local production system; for example, in proximity to fish production areas, direct agreements can be made between schools and producers, or agreements to supply fishing equipment to small-scale fishers can be used to exchange for a portion of the catch (see Box 1). On the other hand, a high acceptance of fish products developed from fish by-products or underutilized, lower-economic value species was demonstrated in a few records, noting that there is greater supply of these as they are traditionally not utilized [67,106].

Box 1 **Improving fish supply chains for HGSF in Cabo Verde [63].**

Started with the support from WFP, the School Canteens Program in Cabo Verde has been in place since 1979. In 2010, as Cabo Verde transitioned to a middle-income country, the school canteens began to be managed 100% by the government. The National School Meal Program had the goal of improving the quality of school meals, raising nutritional awareness through nutrition education, reducing poverty, and stimulating domestic food production.

The pilot project “Local purchases”, implemented from June 2012 to June 2014, aimed at diversifying the school menu, supplying school canteens with national products such as fruits, vegetables, fish, and beans. The project is strengthening linkages with not only local farmers, but also producer organizations and traders, as well as NGOs and civil society organizations. Vegetables and fish are provided, but in much lower quantities than the recommended amount. However, procurement pilot trials for the local procurement of fresh fish from small-scale fisherfolk were initiated with United Nations assistance. There are even a few schools that have a similar arrangement with fishermen. For example, one school assisted a fisherman in purchasing a boat in return for part of his catch.

#### 3.2.5. Good Practices in Food Environments

##### Food Quality and Safety

Guidelines for meal composition and regular monitoring were recommended by two studies [63,105]. A program evaluation from Togo recommended strengthened monitoring, quality and quantity control of school meals by parent–teacher associations, and improved book-keeping for women’s groups responsible for purchasing and preparing foods [105]. In Cabo Verde, it was recommended to define quotas for the supply of local products and support fishing and fish farming organizations in business management, financing for necessary equipment for harvesting, processing and transport, as well as production planning, in order to build organizational capacity and ensure regular supply of fish [63].

##### Promotion, Advertising, and Information of Nutritional Benefits of Fish for School Children

Social behavior change approaches including nutrition education and the promotion of the consumption of fish for children of school age were evidenced as a good practices in order to increase the understanding of healthy eating and meal composition of school meals amongst parents, teachers, and the broader community, and was referced as a good practice in [58]. In addition, an awareness raising on the safety and handling of fish amongst parents, school cooks, teachers, fisherfolk, children, and the broader community also contributed to acceptance of fish in school meals [58].

##### Improved Economic Access through Processing to Reduce Food Loss and Waste

The use of fish by-products and small fish species (see Box 2) was identified as a good practice, as these are generally of a lower economic value, making them more affordable for public procurement programs, and were found to be acceptable to school children [58,66,67,70,84,106,107]. Small fish were commonly utilized whole, in dried or powdered form, and the use of fish by-products processed into a powder allowed for the extension of the amount of fish to serve up to twice as many students when compared to use of fillets only [58]. Three studies demonstrated the high micronutrient content of these products based on whole fish, due to the high concentration of micronutrients in parts (such as eyes, bones, viscera), which are normally wasted [66,67,107].

Box 2 **Repurposing low economic value fish species and by-products for school feeding: a case study from Ghana.**

School feeding has existed in Ghana since 1958, initially implemented by the Catholic Relief Services (CRS) and WFP, with the aim of improving nutrition and tackling poverty in communities [104]. Historically, the food used in school feeding programs consisted of imported food surpluses from the United States of America. However, in 2004, Ghana developed its own national school feeding program, and since 2005, has started to purchase local foods [110].

A pilot study to test the possibility of including fish in school meals utilized factory remnants of tuna frames, in addition to three underutilized fish species (one-man thousand, anchovies, and flying gurnard) which were dried and milled into fish powder and then added to meals for school children [67]. Four local dishes were prepared and assessed by school children for their acceptability based on a hedonic scale—with particularly high scores for anchovies and okra stew, rice with tuna frame powder stew, and rice with flying gurnard stew. Proximate analysis [67] showed that the protein content of all fish powders and the tuna frame powder was high, and nutrient analysis [66] of tuna frames and by-products reflected high iron content. These results demonstrate the potential of low-cost, highly nutritious underutilized fisheries resources and by-products for the nutrient supplementation of traditional dishes while reducing food loss and waste and encouraging sustainable healthy diets.

#### 3.2.6. Good Practices: Consumer Behaviour

In the food system framework, consumer behaviour involves the choice of “where and what food to acquire, prepare, cook, store and eat” [48]. The participatory development of the fish products and recipes for school meals, which included parents, school faculty, fisherfolk, and children themselves was shown to create solutions that responded to the consumers’ (in this case, school children) needs, while also offering derisable and nutritious foods [58,63]. Sensory evaluations of fish products were included in a few studies and were evidenced for ensuring the acceptability of products, thus stimulating demand for these foods in school meals [58,67,70,101,107].

#### 3.2.7. Good Practices: Dietary Quality and Diversity

Improved dietary quality and diversity was noted in a few records which included fish in SFPs, as students who consumed fish had higher dietary diversity scores [74,93]. Another study assessed the dietary diversity of Nigerian school children in eight primary schools, discovering that, while animal-source foods were generally lacking from the diet, fish was the most commonly consumed, and contributed to greater dietary diversity for those who consumed fish [94]. Most studies in this review had a limited consideration of fish as protein-rich foods (along with other animal-source foods and beans), rather than recognizing the multiple micronutrients and fatty acids that fish contribute to diets.

## 4. Discussion

In the reviewed literature, the most researched or evaluated areas of food system entry points for SFPs were food supply chain aspects of production and processing, food environments, consumer behavior and choice, and diets. No studies in this review linked fish consumption in SFPs for school-aged children to nutrition and health outcomes or the broader social, economic and environmental impacts, of food system drivers.

In terms of food supply chains, the source of fish included in SFPs varied from small-scale capture fisheries, small-scale aquaculture, fish by-products, or donations from local parents, community members or international donors, with fish in dried, fresh, or canned form, although many studies did not explicitly state this information. National HGSF programs, such as that of Brazil’s, require a certain percentage of food for school meals to be sourced from local producers [48]; however, these requirements apply to the full basket of foods and not to a percentage of each type of food, thus often resulting in vegetables or other foods purchased from local producers while fish may be sourced in canned form or from imports or donations [97]. As noted in one study [75], caterers sometimes sourced canned fish instead of locally produced fish products due to cost, posing another challenge, as small-scale fisherfolk may be unable to compete with cheap imported or donated canned fish products. The use of fish products processed from by-products is advantageous for the government, as noted in a case from Brazil, where procurement costs reduced and processors increase yield while reducing waste [111]. In Odisha state, India, farming of small, nutritious fish species is encouraged to ensure a regular supply of fish to SFPs [112].

Despite aquaculture now providing more than half of the fish for human consumption globally, its development has been uneven—90% of total aquaculture production globally takes place in Asia, whereas Africa produces less than 2%, half of which is produced in Egypt [113]. Despite this, there are positive examples of the use of aquaculture to provide fish for SFPs. In Kenya, cage culture was used to produce an income for schools to purchase nutritious small fish from capture fisheries for SFPs [84], and in Nigeria, where the HGSF program requires 70 tons of farmed fish per week [89,90].

Population growth in Africa has led to an increasing demand for fish products, resulting in an increased interest in the processing and use of underutilized aquatic species and by-products to combat malnutrition [67,114]. However, considerations for fish handling, storage, and technologies to efficiently process these products, ensuring food safety and consistent supply and access to safe raw materials, are of the utmost importance. Fish by-products, traditionally thrown away, used for livestock, aquaculture or pet feed, fur production or as silage, are regaining attention for human consumption, as they represent a significant source of nutrition while also offering an opportunity to reduce food loss and waste [46,115]. Fish by-products include the head, viscera, skin, bones, and scales, accounting for approximately half of the fish (but a much greater percentage of micronutrients), thus allowing for twice as many servings per whole fish utilized in SFPs. Many fish species—particularly small fish—are already commonly consumed whole (with head, viscera, skin, and bones) in dried or smoked form in SSA countries [50]. Whole small fish and fish by-products can be processed into fish products, such as fish powder, and incorporated or ‘hidden’ in traditional dishes and snacks. A recent study of small fish species common in Ghana [44] complements studies cited in this review [66,67] to show the high nutrient content of small indigenous fish species.

Although no evidence linking fish consumption in SFPs to nutrition outcomes in SSA were found in this review, there is evidence of nutrition and health benefits from trials including fish in high-income countries [26,27,29,115,116,117] for pre-school-aged children [25,26] and school-aged children (although not specifically through SFPs) [28,116,117]. A study including nearly 10,000 students (aged 15 years) in Sweden showed a positive relationship between students who consumed fish at least once per week and higher grades [118]. There is indication that omega-3 fatty acid supplementation and food fortification—such as fish flour spread—hold benefits for cognition in school-age children [101,117,119,120].

The provision of fresh, nutritious foods which are often easily perishable increases the need for safe food handling and hygienic practices [121]. Defining quality standards for food safety and hygiene in every step of the fish supply chain was noted as a necessary step in integrating fish into SFPs—in harvest, transport, fish handling and processing, structure, and equipment in school canteens [63]. The use of fresh or perishable foods requires a functioning cold chain and good hygiene practices to prevent food-borne illness caused by bacterial growth or cross-contamination [121].

Food system entry points for SFPs and HGSF, such as policies and investments and innovation, technology, and infrastructure to support SFPs and local producers in low- and lower-middle income countries are necessary but were not addressed in the literature in this review. Simultaneously, strengthening the capacity of local supply chains and fisheries organizations to sustainably supply safe fish products for SFPs is necessary, and good practices such as supporting small-scale fishers in Cabo Verde (Box 1, [63]) have the potential for scaling. Innovations in technology and infrastructure can help drive the transformation of food systems, particularly in areas that have poor infrastructure and lack cold chain technologies, as addressed in two studies mentioned in this review [58,96]. The majority of fish in Africa are dried and/or smoked in their whole form, using small-scale infrastructure for sun-drying, salting, or smoking the fish [50]. FPC and fish powder are shelf-stable products that can be stored for up to two years in ideal conditions (or three months in local storage regimes), with no need for refrigeration—a great advantage in remote areas without electricity [122,123]. Records identified in this review which incorporated fish powder in SFPs utilized hammer mills at pilot plants connected to research institutions to grind whole small fish or fish by-products into powder [66,67,106].

Creating an enabling environment for small-scale producers and processors can drive more equitable food systems through sociocultural mechanisms. In some of the studies mentioned, processed fish products were highly acceptable; however, there is a need to consider access to equipment and the empowering of small-scale producers, particularly women, as they are more involved in the post-harvest sector of fisheries value chains [46,50]. Small-scale processors may or may not have access to hammer mills which can be used to grind fish (as well as other technologies for drying, smoking, etc.), thus reducing efficiency, compromising the quality of fish products, and increasing time constraints. Hammer mills have been tested in other studies for producing fish powder at the household and community level and have been proven to reduce the time burden for women and food loss when compared to traditional methods, such as mortar and pestle [124]. Ensuring that fish processors have adequate equipment for not only processing but also for transportation is essential, as fish and fish by-products are highly perishable and timely collection is crucial for processing [46]. However, no studies in this review have addressed issues related to transportation and distribution along the supply chains of fish products for SFPs. Building organizational capacity through technical and entrepreneurial training, while also ensuring sustainable sources of funding for small-scale fishing and aquaculture organizations, as well as fish processors’ organizations is integral to ensure a sufficient and consistent supply of safe and good quality fish products for SFPs.

Challenges noted in this review in relation to food availability and access to fish can be dependent on sociocultural constraints (as mentioned above in the context of gender) or can also be driven by environmental constraints. Trends and the seasonality of fish availability are an important consideration when planning HGSF [48], and can vary depending on available fish stocks or seasonality. Fish stocks at higher trophic levels may be less available due to overfishing, while pelagic small fishes such as anchovy and sardine are fished below biologically sustainable levels [46,125]. Pelagic small fish species have higher productivity per unit of biomass than species at higher trophic levels, offering huge potential for a sustainable supply of fish for consumption in SFPs and more broadly [126]; however, they are highly susceptible to climatic changes [46].

To that end, economic drivers and trade-offs between fisheries, aquaculture, agriculture, markets, and development have resulted in some countries prioritizing nutritious wild small fish for trade or animal feeds rather than for human consumption [127,128], which may have a direct impact on the availability and physical access to these nutritious fish for SFPs and for local consumption. Wild small fish are more often produced and traded in low-income countries [129], and while the growth of fish exports from the Global South contributes to an increasing national GDP, it is also criticized for undermining food and nutrition security in developing countries [130,131]. For example, a recent study indicated that some developing countries’ wild catch would be enough to ameliorate micronutrient deficiencies if it was kept for domestic consumption rather than exported [132]. There is no single “answer” to whether small fish should be prioritized for human consumption, as it is necessary to consider the interests of people depending on pelagic fisheries for food and livelihoods, availability, accessibility, and consumer preferences for small fish and other foods [133,134]. To increase access to and desirability of nutritious small fish, there is a need to develop innovative, affordable products from small fish which are easily conserved and accessible to the poor and nutritionally vulnerable [133,134]. The promotion of small fish, by-products, and diverse aquatic foods for human consumption through social protection schemes such as SFPs, can increase food systems resilience [135,136], positively impact ecosystem resilience [125,137], and reduce inequalities in food and nutrition security for those most vulnerable.

Although 49 articles, reports, or evaluations evidencing the inclusion of fish in school feeding programs from 16 countries in SSA were identified for this scoping review, it is important to note that this review was not exhaustive. The list of countries identified may not include all countries in SSA which include fish in their school feeding programs, as the authors were limited by literature that was easily accessible through search terms and by knowledge from key informants on which countries may include fish in their school feeding menus. Further research is needed to explore solutions for identified challenges as well as unexplored food system entry points for the inclusion of fish in SFPs and HGSF. A lack of information on the source of fish included in SFPs and HGSF was apparent in this study, highlighting the need for further investigation into the source of fish in order to gain a better understanding of who benefits from SFPs and how SFPs can shift towards HGSF programs and ensure that the benefits of public procurement are distributed more equitably within local communities. Although there are positive examples from some countries of preferred fish species and acceptance of products made from underutilized species and by-products, this research should be context-specific and adapted to local situations. In addition, training for the development, sensory evaluation, nutrient and contaminant analysis of safe and acceptable fish products, alongside studies assessing the availability and cost of raw materials for SFPs (for example, by using the Fill the Nutrient Gap analysis [138]) is needed in order to expand and scale the use of fish in SFPs.

## 5. Conclusions

A multistakeholder and holistic process engaging ministries of health, fisheries, and education at national, regional, and local levels along with parents, teachers, school canteen cooks, children, and local fisherfolk in the development and testing of fish products for SFPs and HGSF is evidenced as a best practice for developing sustainable, demand-driven interventions. A first step in this process is increasing the recognition and understanding of the nutrient composition of fish extending beyond protein, but for their important contribution to the intake of essential micronutrients and fatty acids. Coupling SFPs with the promotion, information sharing, and awareness raising of the importance of fish in children’s diets is evidenced as a good practice to ensure healthy eating habits for children at school and at home. Regular monitoring of the quantity and quality of fish in school meals can ensure that meal composition and food safety are assured, and defining a minimum percentage of different food items such as fish to be procured from local producers can ensure an equitable distribution of the benefits of HGSF programs for local producers from various sectors. Lastly, product development, sensory evaluation, nutrient and contaminant analysis, and the promotion of nutritional benefits of consumption of fish products produced from underutilized fish—such as pelagic small species—or fisheries by-products can be a cost-effective solution to extend the available supply of fish to feed more students while also reducing food loss, diversifying school menus, and building resilience in the food system.

Enabling conditions within the food environment and a consideration of the drivers impacting the sustainable supply and access to safe, affordable, nutritious fish for nutritionally vulnerable communities are key aspects in shaping programming for SFPs to include fish. The use of diverse, nutritious fish and aquatic foods sourced from small-scale producers and processors can promote ecosystem resilience, resilient communities, and sustainable food systems. Despite this review’s focus on fish in SFPs, it is important to consider fish as part of a diversified diet, along with other nutrient-rich foods. The COVID-19 pandemic has highlighted fragilities in the food system, as well as opportunities for “building forward better”. Adolescence offers a “window of opportunity” for establishing healthy, lifelong nutrition [13] from which children and communities can benefit from through the inclusion of fish in home-grown SFPs.

## Figures and Tables

**Table 1:** Summary of Evidence of fish in SFPs in SSA countries, including FAO classification of Low-Income Food Deficit Countries (LIFDCs) and World Bank country classifications by income.

Country	FAO Classification of LIFDCS (2018)	World Bank Classification (2021)	No. of Records	Reference Numbers
Angola		Lower-Middle Income	1	[58]
Benin	LIFDC	Lower-Middle Income	1	[59]
Burkina Faso	LIFDC	Low-Income	1	[60]
Cape Verde		Lower-Middle Income	3	[61,62,63]
Chad	LIFDC	Low-Income	1	[64]
Cote d’Ivoire	LIFDC	Lower-Middle Income	1	[65]
Ghana	LIFDC	Lower-Middle Income	18	[66,67,68,69,70,71,72,73,74,75,76,77,78,79,80,81,82,83]
Kenya	LIFDC	Lower-Middle Income	2	[84,85]
Mali	LIFDC	Low-Income	1	[86]
Namibia		Upper-Middle Income	1	[87]
Nigeria		Lower-Middle Income	8	[88,89,90,91,92,93,94,95]
Sao Tome and Principe	LIFDC	Lower-Middle Income	1	[96]
Senegal	LIFDC	Lower-Middle Income	3	[97,98,99]
South Africa		Upper-Middle Income	4	[100,101,102,103,104]
Togo	LIFDC	Low-Income	1	[105]
Uganda	LIFDC	Low-Income	2	[106,107]
Total			49	

**Figure A1:**
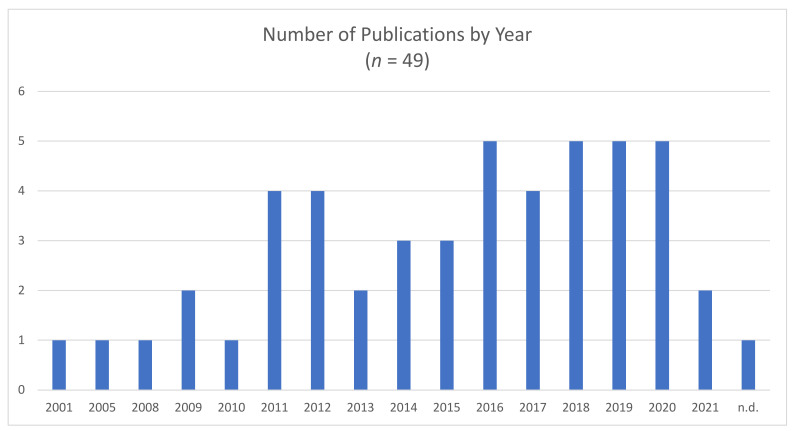
Number of publications by year (2001–2021). One study had no date listed (n.d.).

## Data Availability

The data presented in this study are available in Annex B and summarized in Table 1 of the article.

## Appendix B

**Table A1:** Summary table of records included in the review, including FAO and World Bank country classifications, source and form of fish.

Country	FAO Classification of LIFDCS (2018)	World Bank Classification (2021)	Source of Fish	Form of Fish	Author and Year	Reference Number
Angola		Lower-Middle Income	Small-scale fisheries	Fish powder included in traditional recipes	Toppe et al. forthcoming	[56]
Benin	LIFDC	Lower-Middle Income	Not specified	Fresh then fried	World Bank, 2019	[57]
Burkina Faso	LIFDC	Low-Income	Locally procured (markets)	Unknown	Khachab, 2017	[60]
Cape Verde		Lower-Middle Income	Local producers	“Cooked”	FAO and FAO Representative in Cabo Verde, 2015.	[60]
Cape Verde		Lower-Middle Income	Local producers	Unknown	Silves Ferreira et al., 2018.	[58]
Cape Verde		Lower-Middle Income	Local producers	Unknown	FAO, 2014.	[59]
Chad	LIFDC	Low-Income	Small-scale fisheries (nearby rivers)	Smoked or minced (only flesh) and made into a traditional dumpling with sesame called kanta	Saadie, 2019	[64]
Cote d’Ivoire	LIFDC	Lower-Middle Income	Not specified, mentioned on menu	Dried fish	DNC, PCD and PAM, 2011.	[62]
Ghana	LIFDC	Lower-Middle Income	Low economic value fish and by-products	Fish protein concentrate/fish powder	Ottah Atikpo et al., 2011.	[67]
Ghana	LIFDC	Lower-Middle Income	Low economic value and by-products	Fish protein concentrate/fish powder	Abbey et al., 2017	[63]
Ghana	LIFDC	Lower-Middle Income	Low economic value and by-products	Fish protein concentrate/fish powder	Glover-Amengor et al., 2012	[64]
Ghana	LIFDC	Lower-Middle Income	Source not specified, codfish	Fish protein concentrate/fish powder	Maage et al., 2008	[68]
Ghana	LIFDC	Lower-Middle Income	Not specified	Fish protein concentrate/fish powder	Owusu-Amoako, 2001.	[69]
Ghana	LIFDC	Lower-Middle Income	Not specified	Unknown	Essuman and Bosumtwi-Sam, 2013.	[65]
Ghana	LIFDC	Lower-Middle Income	Not specified	Unknown	Jackson, 2012	[66]
Ghana	LIFDC	Lower-Middle Income	Not specified	small whole fish mashed in soup	Abizari et al., 2014	[106]
Ghana	LIFDC	Lower-Middle Income	Locally procured (markets)	either canned or dried mackerel and cooked into stew	Goldsmith et al., 2019	[84]
Ghana	LIFDC	Lower-Middle Income	Not specified	Dried	Aurino et al., 2020	[76]
Ghana	LIFDC	Lower-Middle Income	Small-scale fisheries	Small whole fish/fish powder	Bigson et al., 2019	[77]
Ghana	LIFDC	Lower-Middle Income	Small-scale fisheries (stocked dams)	Unknown form, cooked into local recipes such as stew	De Carvalho, 2011	[78]
Ghana	LIFDC	Lower-Middle Income	Not specified (study looked at HGSF, but could not assume fish is from local producers)	Tuna flakes, tuna, and/or herrings cooked into various dishes	Kwofie, 2021	[79]
Ghana	LIFDC	Lower-Middle Income	Not specified	Dried fish/anchovies (served whole/dried or fried or in stew)	Owusu J., 2016	[80]
Ghana	LIFDC	Lower-Middle Income	Not specified—fish mentioned in context of some schools reducing fish content of meals due to cost	Unknown	Tagoe, I., 2018.	[81]
Ghana	LIFDC	Lower-Middle Income	Local producers (but also mentions caterers purchasing imported canned fish at times as it was less expensive)	Mentions imported canned fish being substituted for local fish (non-specified form)	Sulemana, 2016	[108]
Ghana	LIFDC	Lower-Middle Income	Local producers	Powdered	Abizari, 2013	[82]
Ghana	LIFDC	Lower-Middle Income	Not specified	Tuna, mackerel and anchovies were the 3 most consumed and they were mainly eaten as boiled, smoked or dried and as protein bases for sauces, soups and stews	Azagba-Nyako, 2017	[83]
Kenya	LIFDC	Lower-Middle Income	Small-scale aquaculture (promotion of products from a project in the area)	Small fish (omena) mentioned in meals but not specific	Nekesa, 2012.	[85]
Kenya	LIFDC	Lower-Middle Income	Aquaculture used to produce income to purchase small fish (dagaa)	Unknown	Van der Knapp and Yongo, 2018.	[70]
Mali	LIFDC	Low-Income	Not specified—fish only mentioned in menu	Fresh/grilled	Cissé, 2012	[86]
Namibia		Upper-Middle Income	Fish donated by parents or community	Unknown form of fish, served with porridge	Kamara, 2016.	[71]
Nigeria		Lower-Middle Income	Aquaculture	Unknown	Falade et al., 2012	[72]
Nigeria		Lower-Middle Income	Small-scale aquaculture	Fresh	Drake et al., 2016	[73]
Nigeria		Lower-Middle Income	Small-scale aquaculture	Unknown	Burbano de Lara, C. 2019.	[74]
Nigeria		Lower-Middle Income	Local producers	Mashed in a soup	Agbon et al., 2012	[83]
Nigeria		Lower-Middle Income	Local producers	Not specified	Igboji et al., 2020	[82]
Nigeria		Lower-Middle Income	Not specified (study looked at HGSF, but could not assume fish is from local producers)	Fresh	Ologele, 2018.	[95]
Nigeria		Lower-Middle Income	Local producers	Fresh, cooked into stew	Adepoju and Johnson. 2020.	[105]
Nigeria		Lower-Middle Income	Not specified	Not specified—dietary recall of children in SFPs	Olatunji, 2015	[107]
Sao Tome and Principe	LIFDC	Lower-Middle Income	Small-scale fisheries	Fresh	Cardoso, and Balde, 2016.	[75]
Senegal	LIFDC	Lower-Middle Income	Donation from Japan	Canned	WFP, Government of Japan. 2020.	[76]
Senegal	LIFDC	Lower-Middle Income	Fish donated by parents or community	Dried	WFP, 2020.	[77]
Senegal	LIFDC	Lower-Middle Income	WFP program but fish provided from parents contributions	Unknown	Diagne et al., 2014	[99]
South Africa		Upper-Middle Income	By-products (fish heads)	Bread spread was developed from fish heads (by-products)	van Stuijvenberg, 2005	[78]
South Africa		Upper-Middle Income	By-products (hake heads) purchased	Fish protein concentrate/fish powder	Dalton et al., 2009	[79]
South Africa		Upper-Middle Income	Unknown source—National School Nutrition Program aims to use school gardens and local production but source of canned fish is not explicit	Canned	Buhl, 2010	[102]
South Africa		Upper-Middle Income	Source of fish not stated	Canned pilchards in tomato sauce	Nhlapo et al., 2015	[103]
South Africa		Upper-Middle Income	Source of fish not stated	Pilchard fish incorporated into South African dishes	Napier et al., 2009	[104]
Togo	LIFDC	Low-Income	Local purchase (aquaculture or fisheries unknown)	Unknown	Andrews et al., 2011	[80]
Uganda	LIFDC	Low-Income	By-products purchased from artisanal processors	Fish protein concentrate/fish powder	FAO, n.d.	[81]
Uganda	LIFDC	Low-Income	Nile Perch (with skin and bones) from Lake Victoria purchased and processed with hammer mill and sieve at research laboratory	Fish powder	Masette et al., 2017	[85]
